# *In Vivo* comparative evaluation of early- and late-stage ACLT-hMnX rat models: a case study of avidin as a cartilage-penetrating nanocarrier for disease-modifying osteoarthritis drugs

**DOI:** 10.3389/fphar.2026.1877598

**Published:** 2026-08-26

**Authors:** Luca Morici, Eric Allémann, Olivier Jordan

**Affiliations:** 1 School of Pharmaceutical Sciences, University of Geneva, Geneva, Switzerland; 2 Institute of Pharmaceutical Sciences of Western Switzerland, Geneva, Switzerland

**Keywords:** anterior cruciate ligament transection with hemi-meniscectomy (ACLT-hMnX), avidin, cartilage regeneration, kartogenin, nanocarrier, osteoarthritis model, protein-based delivery system

## Abstract

**Background:**

Osteoarthritis is the most prevalent joint disease worldwide, affecting over 600 million people. Cartilage-penetrating systems, such as avidin, are emerging as a therapeutic approach for the delivery of disease-modifying osteoarthritis drugs, improving drug uptake and retention in the cartilage, thereby helping to alleviate symptoms and slow disease progression.

**Methods:**

This study aims to investigate whether an avidin-based nanocarrier delivering kartogenin (Av-bKGN) can promote cartilage matrix regeneration in anterior cruciate ligament transection with hemi-meniscectomy (ACLT-hMnX) rat models. We compared outcomes from two ACLT-hMnX rat models of OA which differ in the timing of disease induction, representing the early and late stages of OA.

**Results:**

The results demonstrate that Av-bKGN leads to greater cartilage regeneration compared to the free drug, particularly in the early-stage OA model, whereas the effect is less pronounced in late-stage disease model.

**Conlusion:**

Taken together, these in vivo findings suggest that cartilage-penetrating delivery systems for disease-modifying osteoarthritis drugs are more effective as a preventive strategy in early disease stages rather than as a curative approach in advanced osteoarthritis.

## Graphical Abstract

**Figure:**
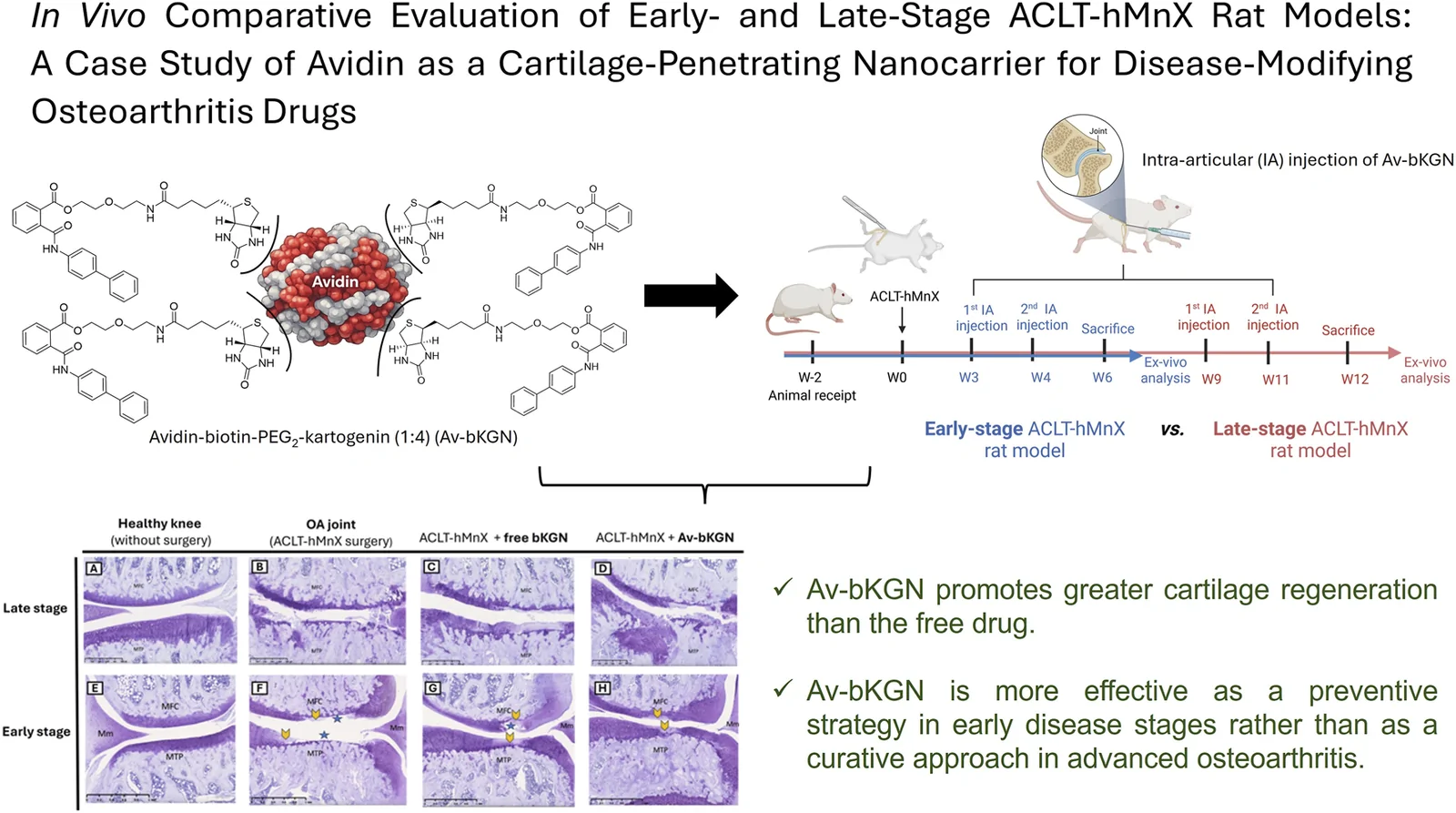

## Highlights

- Avidin-biotinylated kartogenin (Av-bKGN) promotes greater cartilage regeneration compared to the free drug.
- Av-bKGN demonstrates greater efficacy in an early-stage than in an advanced osteoarthritis animal model.
- Cartilage-penetrating delivery systems for DMOADs are more effective as early treatment strategies than as curative approaches.

## 1 Introduction

Osteoarthritis (OA) is the most common joint disease worldwide, affecting over 600 million people (Dell'Isola et al., 2025). The complex physiopathology based on articular cartilage degradation, synovial inflammation and subchondral bone remodeling makes OA challenging to treat. Nowadays only pain and inflammation relief treatments are available to patients while no disease modifying osteoarthritis drugs (DMOADs) capable of restoring cartilage homeostasis have been yet approved by FDA and EMA.

Kartogenin (KGN), first identified in 2012, has been classified as DMOAD for its chondrogenic properties on human mesenchymal stem cells (MSCs) (Johnson et al., 2012). Additionally, KGN has been shown to have chondroprotective effects on human chondrocytes by enhancing extracellular matrix (ECM) synthesis, reducing catabolic activity and promoting cell viability (Morici and Nikolic, 2025). This helps to preserve cartilage integrity. The mechanism of KGN first reported in the literatures by Johnson et al. (2012), related to the disruption of filamin A-CBFβ complex within the chondrocyte and MSC cytoplasm (Marini and Forlino, 2012). KGN interact with the FC-1 of filamin A, a 225-amino acid sequence responsible for interacting with CBFβ; however, the exact binding site and the amino acids involved are yet unknown. The free CBFβ then interact with the runt domain of RUNX1, forming a heterodimer complex. The complex CBFβ-RUNX1 finally internalized in the nucleus mediate the transcription of genes (e.g., SOX-9), inducing chondrogenesis of MSCs into chondrocytes (Morici and Nikolic, 2025). As a result, the amount of aggrecans, collagen type II and metalloproteinase inhibitors increase, and the cartilage ECM is regenerated (Marini and Forlino, 2012). Since the region of CBFβ that interacts with filamin A spans amino acids 68 to 93, the presence of a hydrophobic tryptophan (aa 73) (Yoshida et al., 2005) may play a central role in the interaction with the FC-1 domain of Filamin A, although it has not yet been demonstrated.

KGN is a lipophilic drug of class II BSC with low water solubility (<0.1 mg/mL). One of the challenges in developing DMOADs for clinical use is the difficulty of penetrating and being retained into the cartilage. Following an intra-articular (IA) injection, small free drugs (<10 kDa) remain in the joint space for a short time (a few hours) before being cleared by the lymphatic vessels (Maudens et al., 2018). Over the past few decades, the use of delivery systems such as hyaluronic acid-based hydrogels or nano/micro-particles have been used to prolong drug retention time in the joint (Morici et al., 2025). In our previous work KGN nanocrystals-chitosan microspheres formulated via spray-dryer demonstrated suitable stability and a controlled release profile in synovial fluid, and were found to be non-toxic to human synoviocytes. Nevertheless, due to their large size (∼10 μm), limited cartilage retention was observed (Morici et al., 2023).

Due to the limited porosity of ECM, the conventional sustained-release delivery systems are unable to penetrate trough the different cartilage zones, which has limited the efficacy of DMOADs. To overcome this, a cartilage-penetration strategies are required for DMOADs targeting chondrocytes pathways located deep within the cartilage (Morici et al., 2024). Nanosized carriers, such as cationic peptides and proteins, are emerging as cartilage-penetrating systems based on electrostatic interactions with the anionic proteoglycans of ECM (Morici et al., 2024; Bajpayee et al., 2017a; Hakim et al., 2026).

Avidin is a positively charged glycoprotein originally isolated from egg white and is best known for its exceptionally high affinity for biotin (Kd ∼10^−15^ M) (Green, 1963; Wilchek and Bayer, 1988). Beyond its widespread use in biotechnology and drug delivery applications, avidin possesses physicochemical properties that make it an attractive carrier for targeted intra-articular drug delivery (He et al., 2022a). Due to its small size (approximately 7 nm in diameter) and net positive charge at physiological pH, avidin can effectively penetrate the dense, negatively charged extracellular matrix of articular cartilage through electrostatic interactions with glycosaminoglycans (He et al., 2020). Previous studies have demonstrated that avidin rapidly diffuses throughout the full thickness of cartilage while exhibiting prolonged retention within the tissue, thereby enhancing local drug concentration and reducing systemic exposure (He et al., 2020; Bajpayee et al., 2016). These characteristics have established avidin as a promising cartilage-penetrating nanocarrier for the delivery of therapeutic agents aimed at treating cartilage-related disorders such as OA. *In vivo* studies have demonstrated the potential of avidin as a cartilage-penetrating delivery systems, showing enhanced accumulation and prolonged residence within joint tissues following intra-articular administration (Zhang et al., 2023). A multiarm avidin-based system has been reported to penetrate through the full thickness of rat cartilage within approximately 6 h and to remain detectable in joint tissues for up to 7 days (He et al., 2023). Electrostatic interactions have been shown to govern the rapid cartilage penetration and prolonged retention of intra-articularly injected avidin in rat knee joints, as demonstrated by Bajpayee et al. (2014a). Moreover, avidin-mediated delivery of anti-inflammatory and cartilage-protective agents has been associated with improved therapeutic efficacy, reduced cartilage degeneration, and attenuation of OA progression in preclinical animal models (Bajpayee et al., 2017b). Collectively, these findings support avidin as a promising platform for targeted and sustained intra-articular drug delivery to cartilage.

**FIGURE 1:**
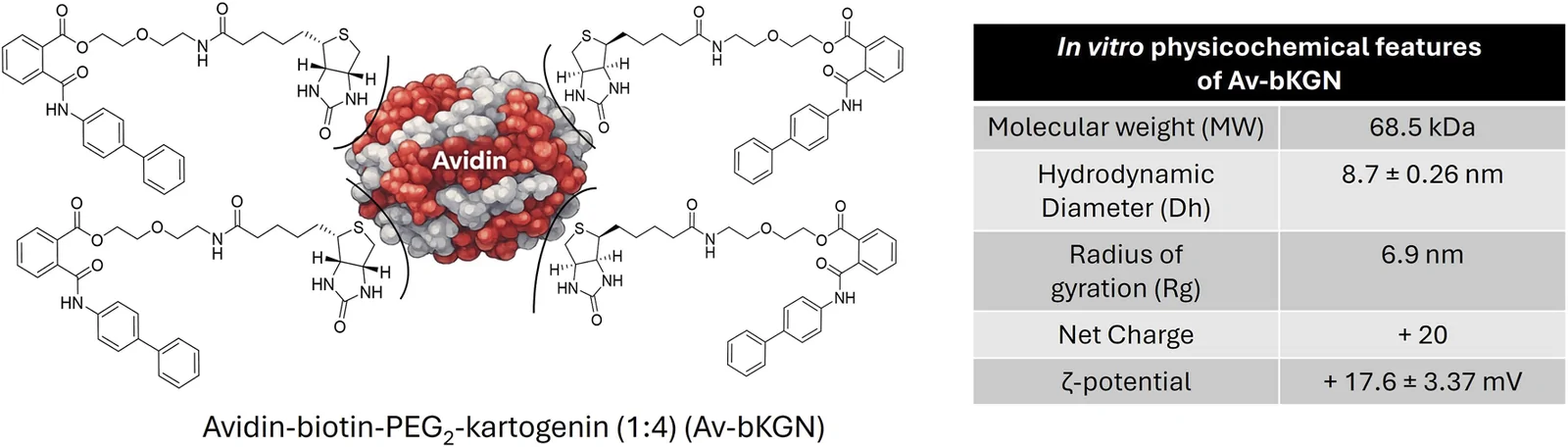
Schematic representation of the tetrameric Av-bKGN with its physicochemical features.

Our recent study showed that biotinized KGN (bKGN) conjugated to avidin (Av-bKGN) improved cartilage uptake and retention in phosphate-buffered saline and simulated synovial fluid compared to the neutral control, neutravidin (Morici et al., 2026). The electrostatic interactions between the cationic Av-bKGN conjugate and the anionic proteoglycan of the extracellular matrix was proven using a diffusivity transport chamber (Morici et al., 2026). Finally, *ex vivo* chondrogenic experiments on IL-1β-inflamed explants showed that a single Av-bKGN dose provided protection equivalent to multiple doses of the free drug, reducing glycosaminoglycans loss by 25%. These results emphasised the potential of electrostatic interaction-driven uptake and retention of Av-bKGN for targeted cartilage therapy. The main physicochemical features of the Av-bKGN nanocarrier are resumed in Figure 1 (Morici et al., 2026).

A critical aspect for advancing preclinical studies lies in the predictive capacity of the chosen animal model (Lon et al., 2023). Cartilage structure, permeability, and ECM composition evolve significantly during OA progression, directly influencing nanocarrier penetration, retention, and therapeutic efficacy (Nikolic and Morici, 2026; Gonzalez-Fernandez et al., 2026). Therefore, selecting an animal model that accurately recapitulates the targeted clinical scenario is essential, not only to demonstrate nanocarrier functionality, but also to ensure that the data obtained are translationally relevant and predictive of human outcomes. In this context, the stage of disease model (early vs. advanced stage OA) is a key determining factor of the observed performance of cartilage-penetrating systems such as Av-bKGN. Our main hypothesis is that advanced-stage OA, characterized by severe ECM degradation, may alter—or potentially limit—the therapeutic effectiveness of avidin-based delivery systems by modifying tissue accessibility and retention dynamics.

The aim of this study is to determine whether the Av-bKGN nanocarrier is capable of inducing cartilage matrix regeneration, by comparing outcomes from two anterior cruciate ligament transection with hemi-meniscectomy (ACLT-hMnX) rat models of OA that differ in the timing of disease induction, representing early vs. late-stage of OA.

## 2 Materials and methods

### 2.1 *In-vivo* study in ACLT-hMnX rat model: early vs. late-stage OA

*In vivo* experiments were performed in collaboration with Atlantic Bone Screen, St-Herblain, France. The animal model used in this study complied with European Directive 2010/63/EU of 2010 and was authorised by the local Ethics Committee and the French Ministry of Education and Research under agreement reference number APAFIS#20051-20190329118075862v7. The main objective of this comparative study was to evaluate the safety and potential efficacy of cartilage matrix regeneration in an OA early and late stage ACLT-hMnX rat model after a double IA injection of avidin nanocarrier (Av-bKGN) at a concentration of 100 µM in 50 µL (∼3,15 µg) per IA injection which correspond to a nominal dose of 10.5 μg/kg. Given the molar ratio of Av:bKGN of 1:4, the concentration of avidin in the formulation was 25 µM in 50 µL (∼330 µg).

### 2.2 Knee osteoarthritis model and surgical procedure

Male Sprague Dawley rats (average weight: 300 g) were housed at the Centre Préclinique Atlanthera (agreement no. A44795175), in compliance with French legislation (decree no. 2013-118, 1 February 2013). The animals were acclimatised for 7–10 days, during which time they were monitored daily for health issues, before the experiments began. They were kept in individually ventilated polypropylene cages (maximum three rats per cage) under controlled temperature and humidity conditions, with a 12-h light/dark cycle. Food and water were provided *ad libitum*.

OA was induced in ten-week-old rats by a surgical procedure performed on the right knee, consisting of an anterior cruciate ligament transection (ACLT) associated with a partial meniscectomy (MnX). Pre-emptive analgesia (buprenorphine/Buprecare® 0.3 mg/ml, Axience, France – 0.05 mg/kg) was injected subcutaneously 30 min before surgery, supplemented by meloxicam (Metacam®/2 mg/ml, Boehringer Ingelheim, Germany). The animals were fully anaesthetised with a mixture of air and isoflurane (air 0.5 L/min–isoflurane 5% Iso-Vet/100%, Piramal, the Netherlands) and maintained under anaesthesia via a flow of air at 0.5 L/min and isoflurane at 2%–3%. They were placed on a warm plate during the surgical procedure. Each animal’s right knee was prepared for surgery and exposed via a para-patellar approach. The skin incision was made laterally to the patella. Some lidocaine (Laocaine®, MSD Santé Animale, France) was applied to the articular capsules immediately before the incision was made. The anterior cruciate ligament was sectioned, and the anterior part of the medial meniscus was resected before the patella was repositioned. The capsules and skin were sutured, and further analgesic injections were administered after the operation and during the following days, depending on the clinical state of the animals that had undergone surgery.

### 2.3 Intra-articular injection of Av-bKGN on ACLT-hMnX model

**FIGURE 2:**
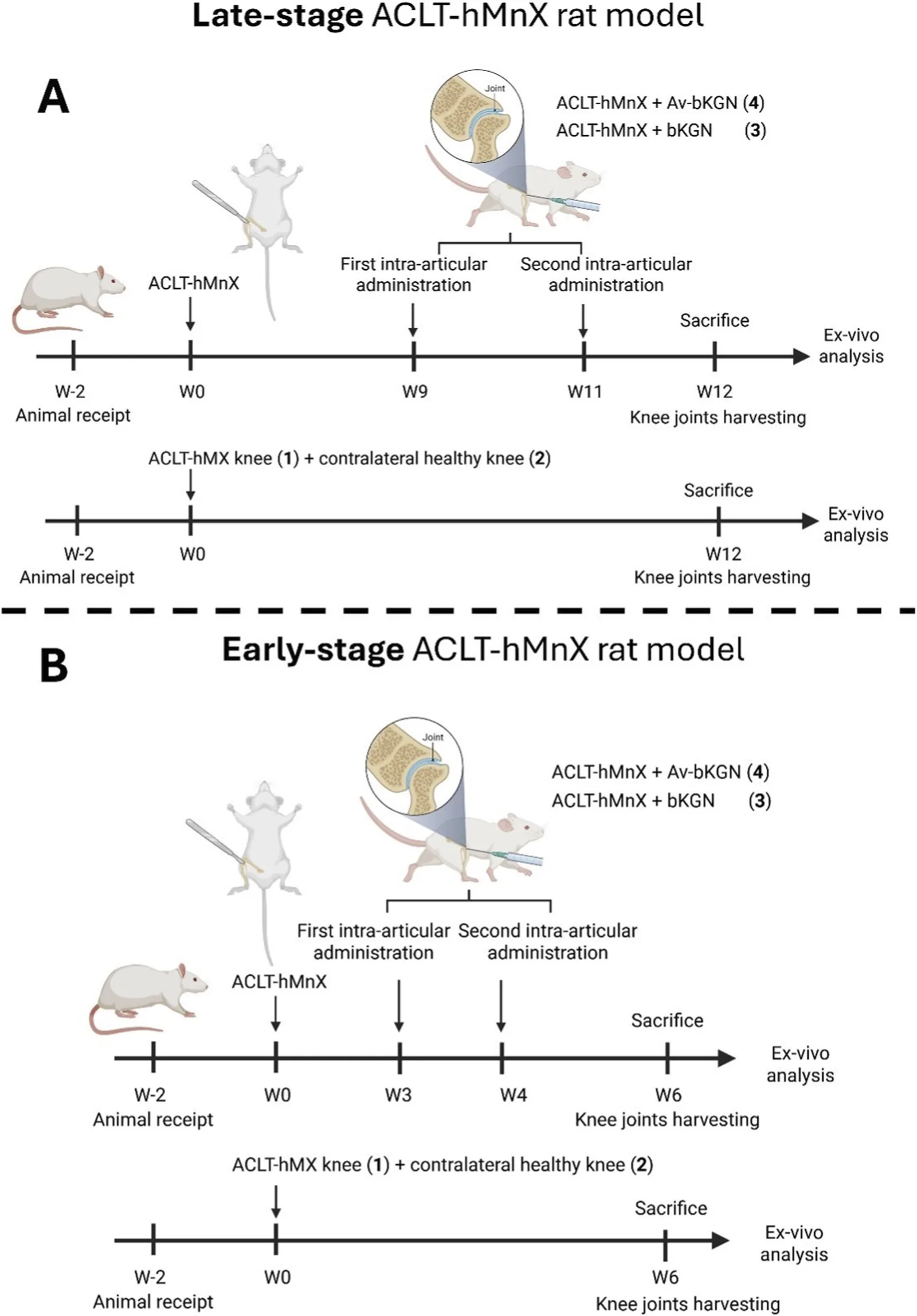
Schematic representation of the late-stage versus early-stage ACLT-hMnX rat model protocol. The conditions investigated were as follows: 1, operated right knees without treatment (control group); 2, contralateral healthy knees of the control group; 3, operated right knee post-treatment bKGN; 4, operated right knee post-treatment Av-bKGN. These conditions were assessed both in the early and in the late-stage models, *n* = 6 animals per condition/group, excepted *n* = 5 for condition 1 at late stage.

bKGN was synthesised and purified according to our previous protocol (Morici et al., 2026). Then bKGN was first dissolved in sterilized DMSO (1 mg/mL) (Sigma Aldrich, US) and conjugated to avidin (Merck KGaA, Germany) in PBS in a molar ratio of 4:1 as reported in our previous study (Morici et al., 2026). Two IA injection of 50 µL of Av-bKGN (100 μM∼ 3.15 μg per injection) were administrated to the operated knees through the patellar ligament using 30-gauge insulin syringes. The rationale for dose selection of 100 μM Av-bKGN is based on the multi-arm avidin nano-construct developed by He et al. (2022b) enables the drug loading content of KGN to be increased to 100 μM maintaining a safe concentration of Av (<1 μM) and effectively suppressing cytokine-induced OA related catabolism-glycosaminoglycans loss. For the late-stage OA model IA injection of Av-bKGN was performed at weeks 9 and 11 post-surgery (Figure 2A). For the OA early-stage model Av-bKGN nanocarrier was administrated intra-articularly at weeks 3 and 4 post-surgery (Figure 2B), on anaesthetised animals via inhalation of an air/isoflurane mixture (air: 0.5 L/min; isoflurane: 2%–3% Iso-Vet 100%, Piramal, the Netherlands). The analgesic protocol (Buprenorphine at 0.05 mg/kg or 166.67 μL/kg by sub cutaneous injections–Buprecare®/0,3 mg/ml, Axience, France) was applied minimum 30 min to 1 h before treatment injection, then between 4-8 h after the first injection. A topical application of lidocaine gel (5%) was applied to the injection site approximately 30 min before the treatment injection. The animals were then placed in a recovery cage to be monitored until recovery. The general clinical state and weight of animals was monitored on a daily basis according to previously defined guidelines (agreement N°APAFIS#47248). Moreover, an in-depth follow-up was performed twice a week for 6 weeks (OA early-stage study) and 12 weeks (OA late-stage study), checking the following criteria: weight, right knee infection and oedema, lameness, articular joint inflammation and global reaction to the treatments (e.g., fever).

The selection of the 6-week (early) and 12-week (late) endpoints was based on the well-established temporal progression of OA in rats (Glasson et al., 2007). The 6-week time point corresponds to the early stage of disease, characterized by mild proteoglycan loss, and initial structural alterations in the articular cartilage, while overall joint architecture remains partially preserved. In contrast, the 12-week time point represents a late-stage osteoarthritic condition, in which more pronounced cartilage erosion, substantial matrix degradation, and established loss of cartilage integrity are typically observed. This stage also includes the development of secondary joint changes such as synovial thickening and subchondral bone remodeling. Taking into consideration the 3R principles, as well as the evidence in the literature demonstrating that avidin has no cartilage regenerative properties (Bajpayee et al., 2014a; Bajpayee et al., 2014b), we decided not to include an additional 12 control rats receiving free avidin in the study for the early- and late-stage models.

### 2.4 Terminal procedure and sampling

The animals were euthanised by cervical dislocation under isoflurane anaesthesia. A local necropsy evaluation was performed by exposing the knee joints (ipsilateral and contralateral). A macroscopic evaluation focusing on local tissue reaction (haematoma/bleeding, inflammatory exudates, fibrous reaction and tissue necrosis) was performed and recorded. The operated knee joints were harvested (*n* = 35) and the contralateral knees for both studies were also harvested for analysis (*n* = 12) as negative control. All 47 samples were immediately stored in formalin solution for a minimum of 72 h and a maximum of 2 weeks to avoid over-fixation and tissue damage that could potentially make subsequent analyses impossible. All sampled knees were decalcified (using EDTA) before trimming, dehydration, and paraffin embedding. Samples were prepared and oriented to be cut (3–5 µm section thickness) in the coronal plane. This orientation allows the exhibition of medial and lateral faces of the femoral condyle and the tibial plateau.

### 2.5 Histological analyses and cartilage/osteophyte evaluation

The thinner location of the meniscus was used to perform TB (Toluidine Blue) histological analysis. Each slide was digitalized with a NanoZoomer S60 (Hamamatsu, Japan) at × 20 magnification to get a global view of the knee joint degradation and a detailed analysis was provided by an independent veterinarian histopathologist of the Deviers-Schorsch Research Pathology (DSRP) using an NDP. View software (Hamamatsu, Japan). The study pathologist was blind to the animal data (rat strain, sex, age) and clinical data. Analyses focused on one slide per group. Cartilage morphology was evaluated through both semi-quantitative evaluation and histomorphometric analyses, specifically targeting the medial tibial plateau and the medial femoral condyle.

#### 2.5.1 Semi-quantitative evaluation

Scores for articular cartilage structure and proteoglycan content were established using a semi-quantitative approach. The semi-quantitative assessment of the parameters articular cartilage structure and proteoglycan content used the OsteoArthritis Research Society International (OARSI) grading scheme, as described for guinea pigs (Kraus et al., 2010). According to the article “The OARSI histopathology initiative -recommendations for histological assessments of osteoarthritis in the rat” written by Gerwin et al. (2010), the modified Mankin score described for guinea pigs may be sufficient for a general histological assessment of OA in rats. In this semi-quantitative approach, the evaluation of the articular cartilage structure and proteoglycan content consists in assigning a grade according to the extension/depth of cartilage matrix loss and the loss of matrix staining, respectively as resumed in Table 1. In addition, the morphological changes in the synovial membrane were evaluated and graded on a scale of 0–4, following the criteria defined by Gerwin et al. (2010) as resumed in Supplementary Table S1.

**TABLE 1:** Parameters used for the semi-quantitative evaluation of cartilage morphology.

Parameter	Grade	Description	Ref
Articular Cartilage Structure	0	Normal, smooth, uninterrupted surface	(Kraus et al., 2010)
	1	Mild surface irregularities (undulations)	
	2	Irregular surface, 1–3 superficial clefts (fissures)	
	3	>3 fissures and/or loss of cartilage in the superficial zone	
	4	1–3 fissures extending into the middle zone	
	5	>3 fissures and/or loss of cartilage extending into the middle zone	
	6	1-3 fissures extending into the deep zone	
	7	>3 fissures extending into the deep zone and/or loss of cartilage to deep zone	
	8	Fissures or loss of cartilage extending to the zone of calcified cartilage	
Proteoglycan Content (staining by toluidine blue)	0	Uniform throughout articular cartilage	(Kraus et al., 2010)
	1	Decreased in superficial zone only and for < half the length of the condyle or plateau	
	2	Decreased in superficial zone for half the length or greater of the condyle or plateau	
	3	Decreased in superficial and middle zones for < half the length of the condyle or plateau	
	4	Decreased in superficial and middle zones for half the length or greater of the condyle or plateau	
	5	Decreased in all 3 zones for < half the length of the condyle or plateau	
	6	Decreased in all 3 zones for half the length or greater of the condyle or plateau	

#### 2.5.2 Histomorphometric analyses

Cartilage morphology was evaluated through histomorphometric analyses, specifically targeting the medial tibial plateau (MTP). Histomorphometric analyses measurements were conducted using the NDP. view software on cartilage matrix loss width at three depths (Gerwin et al., 2010): at the surface (0% depth), at the midzone (50% depth) and at the tidemark (100% depth), by quantify the extent of complete cartilage matrix loss. Areas affected solely by proteoglycan or chondrocyte loss were excluded from this measurement. Cartilage degeneration score was also quantified overall cartilage pathology, encompassing collagen matrix degradation and chondrocyte loss. Areas demonstrating proteoglycan loss alongside chondrocyte death were included, while areas of proteoglycan loss without associated chondrocyte death were excluded. The non-viable cartilage area and the projected cartilage area were delineated, enabling the calculation of a score (on a scale of 0–5) as detailed in Table 2. The overall cartilage degeneration score for MTP was determined via NDP. View software with a maximum score of 15.

**TABLE 2:** Summary of parameters used for histomorphometric analyses.

Parameter	Grade	Description	Ref
Cartilage matrix loss width	-	Width of any cartilage matrix loss along the projected cartilage surface (0% depth), the tidemark (100% depth), and at the midpoint of the cartilage thickness (between surface and tidemark, 50% depth)	(Gerwin et al., 2010)
Cartilage degeneration score	0	No degeneration	(Gerwin et al., 2010)
	1	Minimal degeneration: 5%–10% of the total projected cartilage area affected by matrix or chondrocyte loss	
	2	Mild degeneration: 11%–25% of the total projected cartilage area affected by matrix or chondrocyte loss	
	3	Moderate degeneration: 26%–50% of the total projected cartilage area affected by matrix or chondrocyte loss	
	4	Marked degeneration: 51%–75% of the total projected cartilage area affected by matrix or chondrocyte loss	
	5	Severe degeneration: greater than 75% affected of the total projected cartilage area by matrix or chondrocyte loss	
	Sum	The overall cartilage degeneration score for MTP was determined via NDP.View software with a maximum possible of 15	
Significant cartilage degeneration width	-	Significant: measurement of the width of the articular surface in which 50% or greater of the thickness (from surface to tidemark) is seriously compromised, expressed as a percentage relative to the total width of the articular surface^*^	(Gerwin et al., 2010)
Osteophyte formation	-	Osteophyte formation was evaluated by measuring the largest osteophyte on the medial tibial plateau expressed in micrometre. Marginal zone proliferative changes <200 μm; small 200–299 μm; moderate 300–399 μm; large 400–499 μm; very large >500 μm	(Gerwin et al., 2010)

* The total width of the articular surface is the measured width (µm) of the entire load-bearing cartilage surface in a histological section, used as the reference length for determining the proportion or extent of cartilage degeneration (Gerwin et al., 2010).

Finally, significant cartilage degeneration width was quantified by evaluating the width of cartilage where 50% or more of its thickness (from surface to tidemark) is seriously compromised. It was measured in micrometers using NDP. View software and was reported as a percentage of the overall articular surface width.

Osteophyte formation was evaluated by measuring the largest osteophyte on the medial tibial plateau expressed in micrometre. This measurement was taken from the deepest point of its base at the chondro-osseous junction to the thickest point of the surface of the overlying cartilage, utilizing the NDP. View software.

### 2.6 Statistical analysis

Statistical comparison between the different groups was realized using an ANOVA Two-Way analysis (Tukey’s multiple comparison test) with GraphPad Prism 8.0.1 software (San Diego, CA, United States). Data were reported as the mean ± s.d., considering a 95% confidence interval at significance level *p < 0.05, **p < 0.01, ***p < 0.001, ****p < 0.0001. Groups are resumed as follows for the early-study: *n* = 6 operated right knees without treatment, *n* = 6 contralateral healthy knees of the control group, *n* = 6 operated right knee post-treatment bKGN, *n* = 6 operated right knee post-treatment Av-bKGN. For the late-stage study: *n* = 5 operated right knees without treatment, *n* = 6 contralateral healthy knees of the control group, *n* = 6 operated right knee post-treatment bKGN, *n* = 6 operated right knee post-treatment Av-bKGN.

## 3 Results and discussion

### 3.1 Safety of Av-bKGN in ACLT-hMnX rat model

**FIGURE 3:**
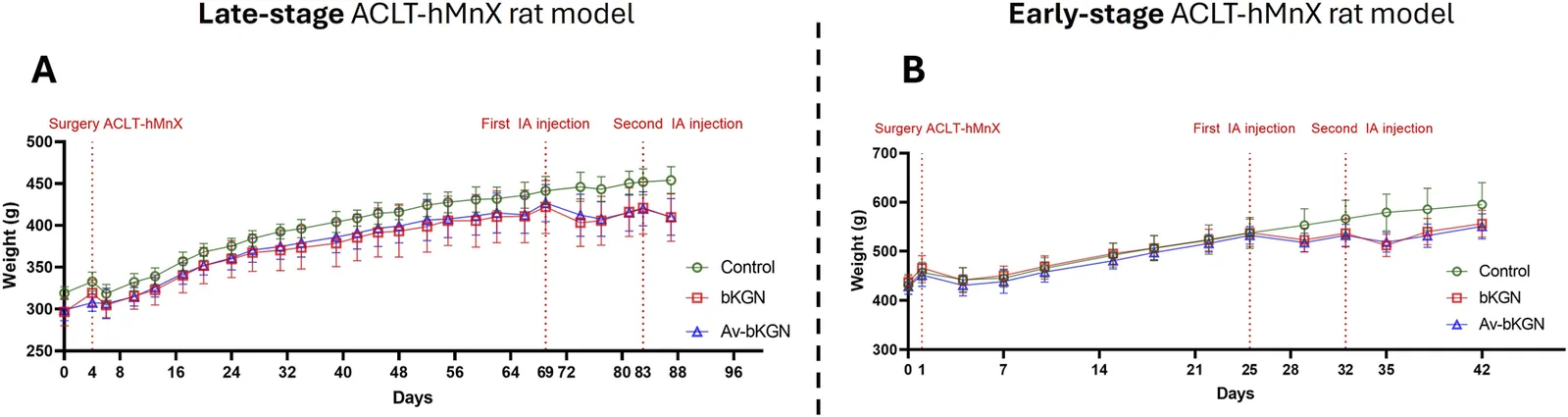
Average rat weight evolution for the **(A)** late-stage versus **(B)** early-stage models.

An increase in animal body weight was observed throughout the *in vivo* phase of the studies, as shown in Figure 3. A slight decrease was observed after the ACLT-hMnX surgery and the IA injections, likely due to antalgic treatment. The general clinical state of the animals was followed twice a week. A few days after the surgery and the IA injections, knee swelling was observed in most cases, with minimal lameness. These swellings disappeared within a few days. Overall, surgery and intra-articular injections were well tolerated by all animals. As body weight is a widely accepted indicator of general systemic toxicity by FDA (International Conference on Harmonisation, 2010), the absence of significant weight loss suggests that the compound was well tolerated and did not produce observable toxicity at the tested dose. These results are consistent with those obtained by Bajpayee et al. (2014a), who demonstrated that avidin doses of up to 1 mM (40X higher than the 25 μM avidin dose injected IA in the present study) were found to be safe and did not affect the viability of bovine cartilage explant cells, nor the catabolism or biosynthesis of the matrix, following IA injection into rat knee joints. Notably, to date, no clinical trials investigating IA injection of avidin in human joints have been reported. However, a phase 3 clinical trial (NCT00311090) has reported that intravenous infusion of 100 mg avidin in a 10 mg/mL solution was safe and well tolerated in both healthy subjects and patients with deep vein thrombosis (DVT) receiving idrabiotaparinux. In that study, avidin rapidly reversed the anti-FXa activity of idrabiotaparinux, demonstrating its neutralising effect (Paty et al., 2010). In the light of these results, further clinical safety dose-response investigations (Phase I) would be required for the topical IA injection of Av-bKGN in OA patients.

### 3.2 Histological and semi-quantitative evaluation of cartilage morphology

**FIGURE 4:**
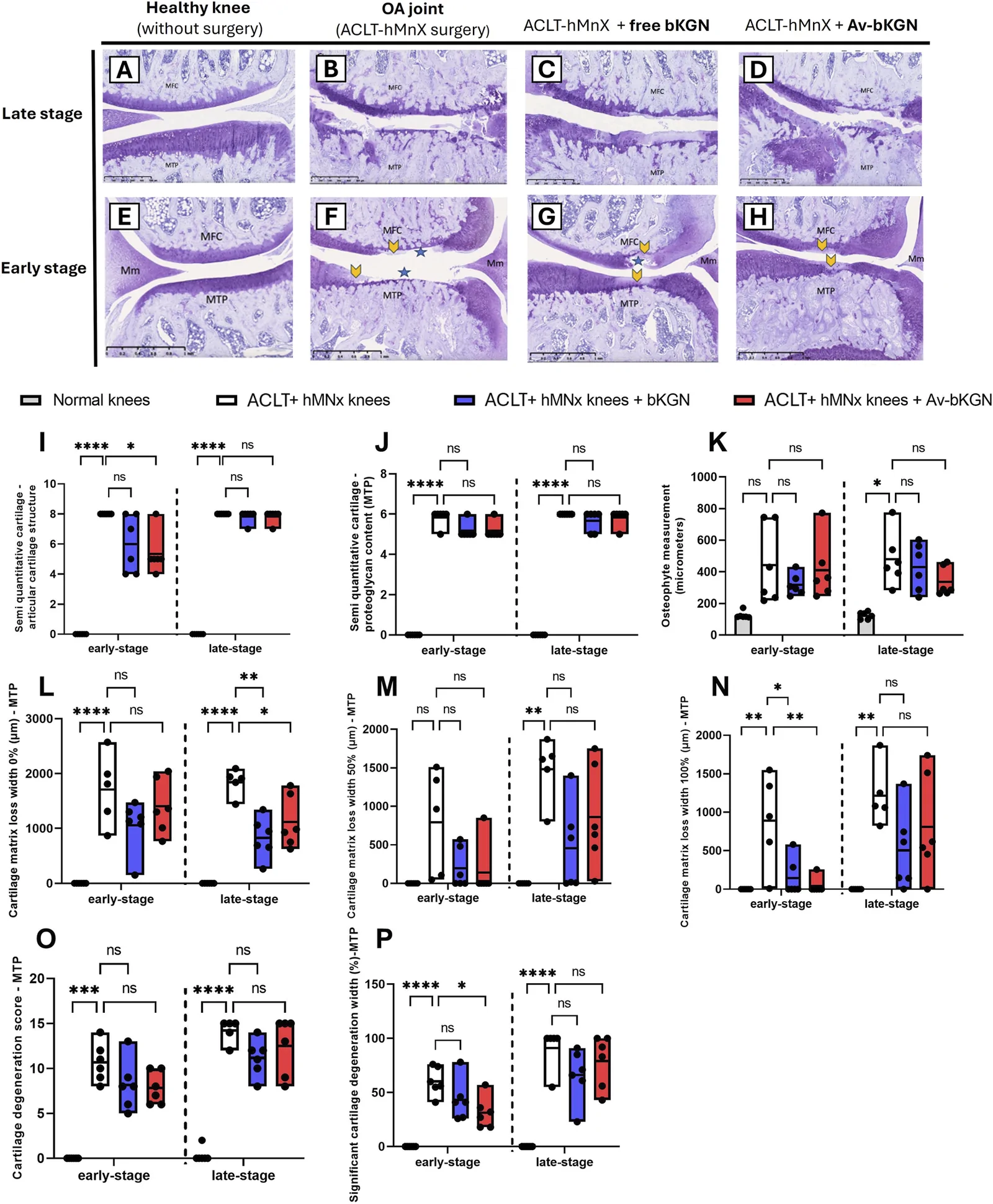
Illustrative pictures, Toluidine Blue-stained sections **(A–H)**. The blue star marks areas of cartilage loss, the yellow arrowhead indicates areas of proteoglycan loss accompanied by chondrocyte depletion. Semi-quantitative evaluation of articular cartilage structure **(I)** and proteoglycan content **(J)**. Histomorphometric evaluation of osteophyte measurement **(K)**, cartilage matrix width at 0% **(L)**, 50% (M), 100% **(N)**, cartilage degeneration score **(O)** and significant cartilage degeneration width of MTP **(P)**. Abbreviations: MFC, Medial Femoral Condyle; Mm, Medial meniscus; MTP, Medial Tibial Plateau.

As illustrated in Figure 4, histopathological evaluation according to the OARSI guidelines confirmed the successful induction of osteoarthritis, as evidenced by the consistent presence of osteoarthritic lesions in all operated knees (Figures 4B,F), whereas non-operated knees (Figures 4A,E) showed no cartilage lesions. The semi-quantitative evaluation of articular cartilage with fissures or loss extending to the calcified cartilage zone (grade 8, Figure 4I), as well as the decreased proteoglycan content in all three zones for half the length or greater of the condyle or plateau (grade 6, Figure 4J), confirmed the successful OA induction in early- and late-stage rat models.

The TB sections of the late-stage model showed no evidence of cartilage matrix regeneration or improvement in the MFC and MTP after two IA injections of free bKGN (Figure 4C) and Av-bKGN (Figure 4D), consistent with the advanced stage of the disease. In contrast, analysis of the TB sections of the early-stage model, indicated that Av-bKGN reduced cartilage loss in both the MTP and the MFC (Figure 4H), whereas bKGN only reduced cartilage loss in the MTP (Figure 4G).

These findings complement the results of the initial OA late-stage study, in which no significant effect on disease progression was observed. The hypothesis raised was that the test items had been evaluated in a curative model, characterized by a more severe and an already established pathology, and that initiating the treatment 9 weeks after ACLT-hMNX surgery was either too late or under-dosed to improve disease progression. In contrast, the present study suggests that a preventive approach, applied during the early stages of osteoarthritis development, could lead to more favorable outcomes. The semi-quantitative evaluation of the early-stage model relative to the OA control knee joint revealed a significant improvement in the structure of the articular cartilage (reduction of fissures and loss of cartilage) following administration of Av-bKGN, with a reduction of the degeneration grade from 8 to around 5. This effect was more pronounced than for free bKGN, for which the grade remained around 6, as shown in Figure 4I. No improvement in articular cartilage structure was observed in the late-stage model, where the degeneration grade remained at 8.

The proteoglycans content (Figure 4J) in both models showed no significant difference compared to the OA joint. In the early-stage model, this finding may be explained by the fact that areas of proteoglycan loss were associated with a depletion of chondrocytes, the cells responsible for proteoglycan synthesis. A limitation of the study is that the rats were sacrificed 2 weeks after the second IA administration of bKGN or Av-bKGN, which may have been too early to observe substantial matrix repair. A longer follow-up period would likely be necessary to allow chondrocytes migration from the calcified cartilage into the newly forming extracellular matrix, thereby promoting renewed proteoglycan production and matrix restoration.

### 3.3 Histomorphometric analyses

Osteophyte formation—a hallmark of chronic and advanced osteoarthritis—requires a longer period to fully develop. Its progressive appearance further supports the distinction between early and late stages of the disease and may explain the significant differences observed between normal control knees and ACLT-injured knees in the late-stage model, as shown in Figure 4K. Osteophyte measurement showed no improvement in the early-stage model (Figure 4K), although a tendency toward reduced osteophyte formation was observed in the late stages following Av-bKGN treatment. Osteophytes are bony outgrowths that develop as part of late-stage OA and involve complex bone/cartilage interactions. Nevertheless, no significant differences were observed in either study. Currently, there is no clear, direct evidence in the published literature demonstrating that KGN prevents or reduces osteophyte formation in osteoarthritis models.

The histomorphometric analysis of cartilage matrix loss in the early-stage model at 0% and 50% cartilage width showed no significant difference between OA joints and the two treatment groups, bKGN and Av-bKGN (Figures 4L,M). In contrast, significant differences were observed at 100% cartilage width (Figure 4N). Specifically, the cartilage matrix loss width was reduced from 892 μm in the OA joint group to 116 μm (∼8-fold reduction) in the bKGN group, and to 51 μm (∼17-fold reduction) in the Av-bKGN group. These results confirmed that the treated groups showed slightly less advanced cartilage lesions in the MTP compared with the operated knees of the control group. This was further supported by lower cartilage degeneration scores (trend towards an increased efficacy using Av-bKGN, see Figure 4O), as well as reduced significant degeneration widths (significantly lower for Av-bKGN, see Figure 4P). In particular, the significant cartilage degeneration width was reduced by half using Av-bKGN, decreasing from 60% in the OA (ACLT + hMnX) group to around 31%. No significant difference was observed following treatment with the free drug bKGN (see Figure 4P). This could be explained by the cartilage-penetrating properties of avidin-based delivery systems, as reported in previous studies (He et al., 2020; Morici et al., 2026; Bajpayee et al., 2014b; Wagner et al., 2020). In fact, avidin has demonstrated an increased ability to be taken up and retained in bovine cartilage explants, driven by electrostatic interactions with the anionic components (GAGs) of the extracellular matrix. Moreover, Av-bKGN has demonstrated promising drug depot capabilities, ensuring the protection of the cartilage matrix under IL-1α induced OA-simulated conditions (Morici et al., 2026).

These results contrast with the initial findings of the OA late-stage model, in which no significant effect on the development of OA was observed between the two treatment groups and the ACLT + hMnX surgery group, except at 0% width (Figure 4L), where both treatment groups reduced the width of cartilage matrix loss from around 1844 μm (OA group) to 1,000 μm (bKGN) and 1,021 μm (Av-bKGN) (a 1.8-fold reduction for both treatment groups). Again, no significant difference was observed between the OA group and the treated groups in terms of cartilage degeneration score (Figure 4O) or significant cartilage degeneration width (Figure 4P). It is important to note that the late-stage ACLT + hMnX model represent an advanced and aggressive form of OA, characterized by an almost complete cartilage matrix degradation. A cartilage degeneration score of 15 for ACLT + hMnX surgery late-stage model (Figure 4O) confirms severe degeneration, with more than 75% of the total projected cartilage area affected by matrix or chondrocyte loss. In comparison, the early-stage model exhibited a degeneration score of approximately 10 corresponding to a moderate to marked degeneration with 26%–75% of the total projected cartilage area affected by matrix or chondrocyte loss.

As shown in Figure 4P, the cartilage degeneration width reached 100% in the ACLT + hMnX late-stage group compared to approximately 60% in the early-stage group, further confirming that the disease model was extremely advanced. Under such severe conditions, the near-complete loss of extracellular matrix likely limited the chondroprotective and regenerative efficacy of both bKGN and Av-bKGN, as the presence of a residual ECM scaffold appears necessary for therapeutic response/efficacy.

It was therefore hypothesized that the late-stage OA model represent a curative setting, more severe and characterized by extended pathological damages, in which initiating the treatment 9 weeks after the ACLT-hMnX surgery turned it inefficient to slow down disease progression. In contrast, the early-stage study suggests that a preventive approach, applied during the initial phases of OA development, may lead to improved outcomes. Overall, this study demonstrates that DMOADs such as KGN, when formulated in a way they can effectively reach and accumulate within the cartilage, offer an innovative approach to treat osteoarthritis. Although the benefits observed here were modest—particularly in terms of cartilage lesion reduction—the findings suggest that an early, preventive administration may hold greater promise than curative strategies applied at later stages. These results highlight the importance of treatment timing in modulating disease progression.

The semi-quantitative evaluation of the synovial membrane is presented in Supplementary Figure S1. In the non-operated control group (Normal knee, Group 1), no synovial membrane inflammation was observed. In the operated groups (ACLT-hMnX, Group 2), histopathological evaluation revealed synovial membrane alterations comparable to those observed in the bKGN- and Av-bKGN-treated groups, with synovial membrane inflammation scores ranging from 2 to 4. These alterations were characterized by moderate to marked fibrosis in the subsynovial tissue along with slight inflammatory cell infiltration across all operated and treated groups.

The semi-quantitative synovial membrane evaluation did not reveal any significant impact of the treatment. A more rigorous assessment of inflammation would require the use of dedicated inflammatory models, such as collagen-induced arthritis (CIA) or monoiodoacetate-induced arthritis (MIA), combined with the measurement of quantitative markers such as pro-inflammatory cytokines level (e.g., IL-1β, IL-6).

To further refine the *in vivo* protocol, larger animal models—such as the ACLT rabbit model (Bajpayee et al., 2017b; Boyer et al., 2025)—can be employed, as it has been shown that cartilage thickness influences intra-articular drug delivery and retention within cartilage (Bajpayee et al., 2015). This model offers a valuable platform for evaluating cartilage-targeting cationic peptide and protein carriers (Bajpayee et al., 2014b). Notably, the joint anatomy, cartilage morphology, and biomechanical properties in larger animals more closely resemble those of humans (Dou et al., 2023), making them a more clinically relevant model for translational studies.

The decision not to include an additional vehicle-only control group (avidin alone) was based on the fact that avidin is not a biologically active cartilage repair factor. Previous studies have demonstrated that avidin does not modulate chondrocyte viability, extracellular matrix synthesis, or glycosaminoglycan loss when administered alone, supporting its role as an inert delivery carrier rather than a therapeutic agent. Furthermore, the *in vivo* immunogenicity profile of avidin has already been evaluated and shown to be acceptable at the concentrations used in similar studies (Bajpayee et al., 2014a).

Future studies involving larger sample sizes and longer follow-up periods are warranted to obtain more robust data. Investigations into the use of Av-bKGN combined with factors that could potentiate its effects—particularly on cartilage regeneration and its anti-inflammatory properties, which were not evident at the synovial membrane level in this study—could provide valuable insights and enhance the efficacy of preventive treatments. Further micro-CT analysis investigating subchondral bone remodeling in late-stage OA models treated with Av-bKGN are warranted, as kartogenin has been reported in the literature to reduce bone turnover and to prevent subchondral bone changes in the ACLT rat model (Mohan et al., 2016).

Due to the complexity of the intra-articular environment, including synovial fluid turnover, lymphatic drainage, and OA-associated pathological changes such as immune cell accumulation, the *in vivo* retention half-life of Av-bKGN remains challenging to quantify. Furthermore, the very limited volume (microliter scale) of synovial fluid in rat knee joints, which may be substantially reduced or absent following ACLT surgery, makes direct measurement of intra-articular retention particularly challenging and represents an inherent limitation of rodent models for IA administration. An *In Vivo* Imaging System (IVIS)-based approach using fluorescently labeled avidin (e.g., avidin-FITC) could provide an alternative method to evaluate joint retention; however, fluorescent labeling may alter the physicochemical properties of the carrier and potentially affect its *in vivo* behavior (Álamo et al., 2020) and cartilage penetration characteristics. Therefore, future studies may investigate the intra-articular retention, systemic and lymphatic biodistribution, and interactions of Av-bKGN with immune components in larger animal models to better characterize its pharmacokinetic profile and therapeutic potential.

Another limitation of this study is that the analysis was restricted to a single histological section per knee, in contrast to OARSI recommendations which call for the examination of multiple sections throughout the entire joint in order to accurately assess the most severely affected areas. Our histological findings should be interpreted as representative of the standardized sampling site rather than the entire joint. Finally, the duration of the early-stage model was half that of the late-stage model (12 weeks). Extending the early-stage study to 12 weeks may have produced more reliable results, since new ECM is already produced by week 6 (Figures 4G,H) in this model.

Overall, integrating a cartilage-penetrating delivery system such as avidin with kartogenin may potentiate its therapeutic efficacy, representing a promising and innovative strategy for the treatment of early-stage OA.

## 4 Conclusion

An initial *in vivo* campaign using a late-stage OA model did not yield significant result after two intra-articular injections of Av-bKGN administered at week 9 and 12 post-surgery. Therefore, an early OA stage model was selected, with two intra-articular injections of Av-bKGN at weeks 3 and 4 post-surgery with the aim to prevent OA evolution. In this latter model, the scores for articular cartilage damage were significantly better for the avidin nanocarrier compared to the free drug. Histomorphometric analysis confirmed that the treated groups (bKGN and Av-bKGN) showed less advanced cartilage lesions in the MTP compared to the operated knees of the control group. This was evidenced by lower cartilage degeneration scores (a trend observed for Av-bKGN), reduced cartilage matrix loss – particularly at 100% of the cartilage width (significantly improved for Av-bKGN), and smaller degeneration widths (significantly improved for Av-bKGN). Interestingly, analysis of the TB-stained histological sections demonstrated Av-bKGN reduced cartilage loss in both the MTP and the MFC, whereas bKGN only reduced cartilage loss in the MTP. These results collectively suggest that the administration of an avidin nanocarrier in combination with a DMOAD such as kartogenin may enhance its therapeutic efficacy.

## Data Availability

The raw data supporting the conclusions of this article will be made available by the authors, without undue reservation.

## References

[B1] Álamo P. Pallarès V. Céspedes M. V. Falgàs A. Sanchez J. M. Serna N. (2020). Fluorescent dye labeling changes the biodistribution of tumor-targeted nanoparticles. Pharmaceutics 12, 1004. 10.3390/pharmaceutics12111004 33105866 PMC7690626

[B2] Bajpayee A. G. Scheu M. Grodzinsky A. J. Porter R. M. (2014a). Electrostatic interactions enable rapid penetration, enhanced uptake and retention of intra-articular injected avidin in rat knee joints. J. Orthop. Res. 32, 1044–1051. 10.1002/jor.22630 24753019

[B3] Bajpayee A. G. Wong C. R. Bawendi M. G. Frank E. H. Grodzinsky A. J. (2014b). Avidin as a model for charge driven transport into cartilage and drug delivery for treating early stage post-traumatic osteoarthritis. Biomaterials 35, 538–549. 10.1016/j.biomaterials.2013.09.091 24120044 PMC3863604

[B4] Bajpayee A. G. Scheu M. Grodzinsky A. J. Porter R. M. (2015). A rabbit model demonstrates the influence of cartilage thickness on intra-articular drug delivery and retention within cartilage. J. Orthop. Res. 33, 660–667. 10.1002/jor.22841 25627105

[B5] Bajpayee A. G. Quadir M. A. Hammond P. T. Grodzinsky A. J. (2016). Charge based intra-cartilage delivery of single dose dexamethasone using avidin nano-carriers suppresses cytokine-induced catabolism long term. Osteoarthr. Cartil. 24, 71–81. 10.1016/j.joca.2015.07.010 26211608 PMC4695287

[B6] Bajpayee A. G. Grodzinsky A. J. (2017a). Cartilage-targeting drug delivery: can electrostatic interactions help? Nat. Rev. Rheumatol. 13, 183–193. 10.1038/nrrheum.2016.210 28202920

[B7] Bajpayee A. G. De la Vega R. E. Scheu M. Varady N. H. Yannatos I. A. Brown Y. K. L. A. (2017b). Sustained intra-cartilage delivery of low dose dexamethasone using a cationic carrier for treatment of post traumatic osteoarthritis. Eur. Cell. Mater. 34, 341–364. 10.22203/eCM.v034a21 29205258 PMC5744663

[B8] Boyer T. L. Chao O. Hakim B. Childress L. Meslier Q. A. Iyengar S. M. (2025). Cartilage targeting cationic peptide carriers display deep cartilage penetration and retention in a rabbit model of post-traumatic osteoarthritis. Osteoarthr. Cartil. 33, 721–734. 10.1016/j.joca.2025.04.001 40222626 PMC12242911

[B9] Dell'Isola A. Recenti F. Giardulli B. Lawford B. J. Kiadaliri A. (2025). Osteoarthritis year in review 2025: epidemiology and therapy. Osteoarthr. Cartil. 33, 1300–1306. 10.1016/j.joca.2025.08.015 40914550

[B10] Dou H. Wang S. Hu J. Song J. Zhang C. Wang J. (2023). Osteoarthritis models: from animals to tissue engineering. J. Tissue Eng. 14, 20417314231172584. 10.1177/20417314231172584 37223125 PMC10201005

[B11] Gerwin N. Bendele A. M. Glasson S. Carlson C. S. (2010). The OARSI histopathology initiative - recommendations for histological assessments of osteoarthritis in the rat. Osteoarthr. Cartil. 18 (Suppl. 3), S24–S34. 10.1016/j.joca.2010.05.030 20864021

[B12] Glasson S. S. Blanchet T. J. Morris E. A. (2007). The surgical destabilization of the medial meniscus (DMM) model of osteoarthritis in the 129/SvEv mouse. Osteoarthr. Cartil. 15, 1061–1069. 10.1016/j.joca.2007.03.006 17470400

[B13] Gonzalez-Fernandez P. Morici L. Simula L. Takwa R. Benabdellah A. Allémann E. (2026). Cationic liposome-mediated intra-articular delivery of lorecivivint for osteoarthritis treatment. Eur. J. Pharm. Sci. 220, 114980. 10.1016/j.ejpb.2026.114980 41519310

[B14] Green N. M. (1963). AVIDIN. 1. THE USE OF (14-C)BIOTIN FOR KINETIC STUDIES AND FOR ASSAY. Biochem. J. 89, 585–591. 10.1042/bj0890585 14101979 PMC1202466

[B15] Hakim B. Zhang H. Selvadoss A. Bajpayee A. (2026). Peptide-based targeted drug delivery strategies for osteoarthritis treatment. NPJ Biomed. Innov. 3, 27. 10.1038/s44385-026-00082-w 42032264 PMC13065784

[B16] He T. Zhang C. Vedadghavami A. Mehta S. Clark H. A. Porter R. M. (2020). Multi-arm avidin nano-construct for intra-cartilage delivery of small molecule drugs. J. Control. Release 318, 109–123. 10.1016/j.jconrel.2019.12.020 31843642 PMC7424591

[B17] He T. Zhang C. Bajpayee A. G. (2022a). Charge-based multiarm avidin nanoconstruct as a platform technology for applications in drug delivery. Methods Mol. Biol. 2, 537–553. 10.1007/978-1-0716-1811-0_28 35094345

[B18] He T. Shaw I. Vedadghavami A. Bajpayee A. G. (2022b). Single-dose intra-cartilage delivery of kartogenin using a cationic multi-arm avidin nanocarrier suppresses cytokine-induced osteoarthritis-related catabolism. Cartilage 13, 19476035221093072. 10.1177/19476035221093072 35491681 PMC9251829

[B19] He T. Zhang C. Colombani T. Bencherif S. A. Porter R. M. Bajpayee A. G. (2023). Intra-articular kinetics of a cartilage targeting cationic PEGylated protein for applications in drug delivery. Osteoarthr. Cartil. 31, 187–198. 10.1016/j.joca.2022.09.010 36241136 PMC9892226

[B20] International Conference on Harmonisation (2010). Guidance on M3(R2) nonclinical safety studies for the conduct of human clinical trials and marketing authorization for pharmaceuticals; availability. Not. Fed. Regist. 75, 3471–3472. 20349552

[B21] Johnson K. Zhu S. Tremblay M. S. Payette J. N. Wang J. Bouchez L. C. (2012). A stem cell–based approach to cartilage repair. Science 336, 717–721. 10.1126/science.1215157 22491093

[B22] Kraus V. B. Huebner J. L. DeGroot J. Bendele A. (2010). The OARSI histopathology initiative - recommendations for histological assessments of osteoarthritis in the guinea pig. Osteoarthr. Cartil. 18, S35–S52. 10.1016/j.joca.2010.04.015 20864022 PMC2948547

[B23] Longo U. G. Papalia R. De Salvatore S. Picozzi R. Sarubbi A. Denaro V. (2023). Induced models of osteoarthritis in animal models: a systematic review. Biology 12, 283. 10.3390/biology12020283 36829562 PMC9953428

[B24] Marini J. C. Forlino A. (2012). Replenishing cartilage from endogenous stem cells. N. Engl. J. Med. 366, 2522–2524. 10.1056/NEJMcibr1204283 22738103 PMC6320687

[B25] Maudens P. Jordan O. Allémann E. (2018). Recent advances in intra-articular drug delivery systems for osteoarthritis therapy. Drug Discov. Today 23, 1761–1775. 10.1016/j.drudis.2018.05.023 29792929

[B26] Mohan G. Magnitsky S. Melkus G. Subburaj K. Kazakia G. Burghardt A. J. (2016). Kartogenin treatment prevented joint degeneration in a rodent model of osteoarthritis: a pilot study. J. Orthop. Res. 34, 1780–1789. 10.1002/jor.23197 26895619 PMC6348064

[B27] Morici L. Nikolic I. (2025). Update on clinical trials of intra-articular disease-modifying osteoarthritis drugs inducing cartilage regeneration. Eur. J. Pharm. Sci. 217, 107403. 10.1016/j.ejps.2025.107403 41371377

[B28] Morici L. Gonzalez-Fernandez P. Jenni S. Porcello A. Allémann E. Jordan O. (2023). Nanocrystal-chitosan particles for intra-articular delivery of disease-modifying osteoarthritis drugs. Int. J. Pharm. 651, 123754. 10.1016/j.ijpharm.2023.123754 38163526

[B29] Morici L. Allémann E. Rodríguez-Nogales C. Jordan O. (2024). Cartilage-targeted drug nanocarriers for osteoarthritis therapy. Int. J. Pharm. 666, 124843. 10.1016/j.ijpharm.2024.124843 39424088

[B30] Morici L. Jordan O. Allémann E. R.-N. C. Rodríguez-Nogales C. (2025). Recent advances in nanocrystals for arthritis drug delivery. Expert Opin. Drug Deliv. 22 (7), 1031–1042. 10.1080/17425247.2025.2505758 40357685 PMC12312752

[B31] Morici L. Jenni S. Hakim B. Rodríguez-Nogales C. Allémann E. Bajpayee A. G. (2026). Protein-based nanocarrier delivering kartogenin derivative to cartilage matrix for intra-articular treatment of osteoarthritis. J. Drug Deliv. Sci. Technol. 119, 108162. 10.1016/j.jddst.2026.108162 42305633 PMC13267916

[B32] Nikolic I. Morici L. (2026). When healing turns fibrotic: exploring molecular mechanisms and therapeutic strategies for knee arthrofibrosis. Eur. J. Pharmacol. 1017, 178645. 10.1016/j.ejphar.2026.178645 41655683

[B33] Paty I. Trellu M. Destors J. M. Cortez P. BoËLle E. Sanderink G. (2010). Reversibility of the anti-FXa activity of idrabiotaparinux (biotinylated idraparinux) by intravenous avidin infusion. J. Thromb. Haemost. 8, 722–729. 10.1111/j.1538-7836.2010.03746.x 20088937

[B34] Wagner E. K. Vedadghavami A. Jacobsen T. D. Goel S. A. Chahine N. O. Bajpayee A. G. (2020). Avidin grafted dextran nanostructure enables a month-long intra-discal retention. Sci. Rep. 10, 12017. 10.1038/s41598-020-68351-1 32694557 PMC7374582

[B35] Wilchek M. Bayer E. A. (1988). The avidin-biotin complex in bioanalytical applications. Anal. Biochem. 171, 1–32. 10.1016/0003-2697(88)90120-0 3044183

[B36] Yoshida N. Ogata T. Tanabe K. Li S. Nakazato M. Kohu K. (2005). Filamin A-bound PEBP2beta/CBFbeta is retained in the cytoplasm and prevented from functioning as a partner of the Runx1 transcription factor. Mol. Cell. Biol. 25, 1003–1012. 10.1128/MCB.25.3.1003-1012.2005 15657428 PMC543995

[B37] Zhang C. Vedadghavami A. He T. Charles J. F. Bajpayee A. G. (2023). Cationic carrier mediated delivery of anionic contrast agents in low doses enable enhanced computed tomography imaging of cartilage for early osteoarthritis diagnosis. ACS Nano 17, 6649–6663. 10.1021/acsnano.2c12376 36989423 PMC10629240

