# Supplementary material for: In Vivo comparative evaluation of early- and late-stage ACLT-hMnX rat models: a case study of avidin as a cartilage-penetrating nanocarrier for disease-modifying osteoarthritis drugs
Source: Front Pharmacol. 2026 Aug 26;17:1877598. doi: 10.3389/fphar.2026.1877598 (PMC13553433; doi:10.3389/fphar.2026.1877598)
Supplement: Supplementary file 1 [file DataSheet1.pdf]

Supplementary material

In Vivo Comparative Evaluation of Early- and Late-Stage ACLT-hMnX Rat Models: A Case Study of Avidin as a Cartilage-Penetrating Nanocarrier for Disease-Modifying Osteoarthritis Drugs

Luca Morici[1,2*], Eric Allémann[1,2], Olivier Jordan[1,2*]

[1] School of Pharmaceutical Sciences, University of Geneva, Rue Michel-Servet 1, 1211 Geneva 4, Switzerland

[2]Institute of Pharmaceutical Sciences of Western Switzerland, Rue Michel-Servet 1, 1211 Geneva 4, Switzerland

*** Correspondence:**
Corresponding Authors
luca.morici@unige.ch; olivier.jordan@unige.ch

S1. Semi-quantitative evaluation of synovial membrane

TableS1: The morphological changes in the synovial membrane were evaluated and graded on a scale of 0 to 4, following the criteria defined by Gerwin et *al.*[30].

Parameter	Grade	Description
Synovial membrane inflammation score	0	No changes (1-2 layers of synovial lining cells).
	1	Increased number of lining cell layers (3-4 layers) or slight proliferation of subsynovial tissue.
	2	Increased number of lining cell layers (3-4 layers) and/or proliferation of subsynovial tissue.
	3	Increased number of lining cell layers (>4 layers) and/or proliferation of subsynovial tissue and infiltration of few inflammatory cells.
	4	Increased number of lining cell layers (>4 layers) and/or proliferation of subsynovial tissue, infiltration of large number of inflammatory cells.

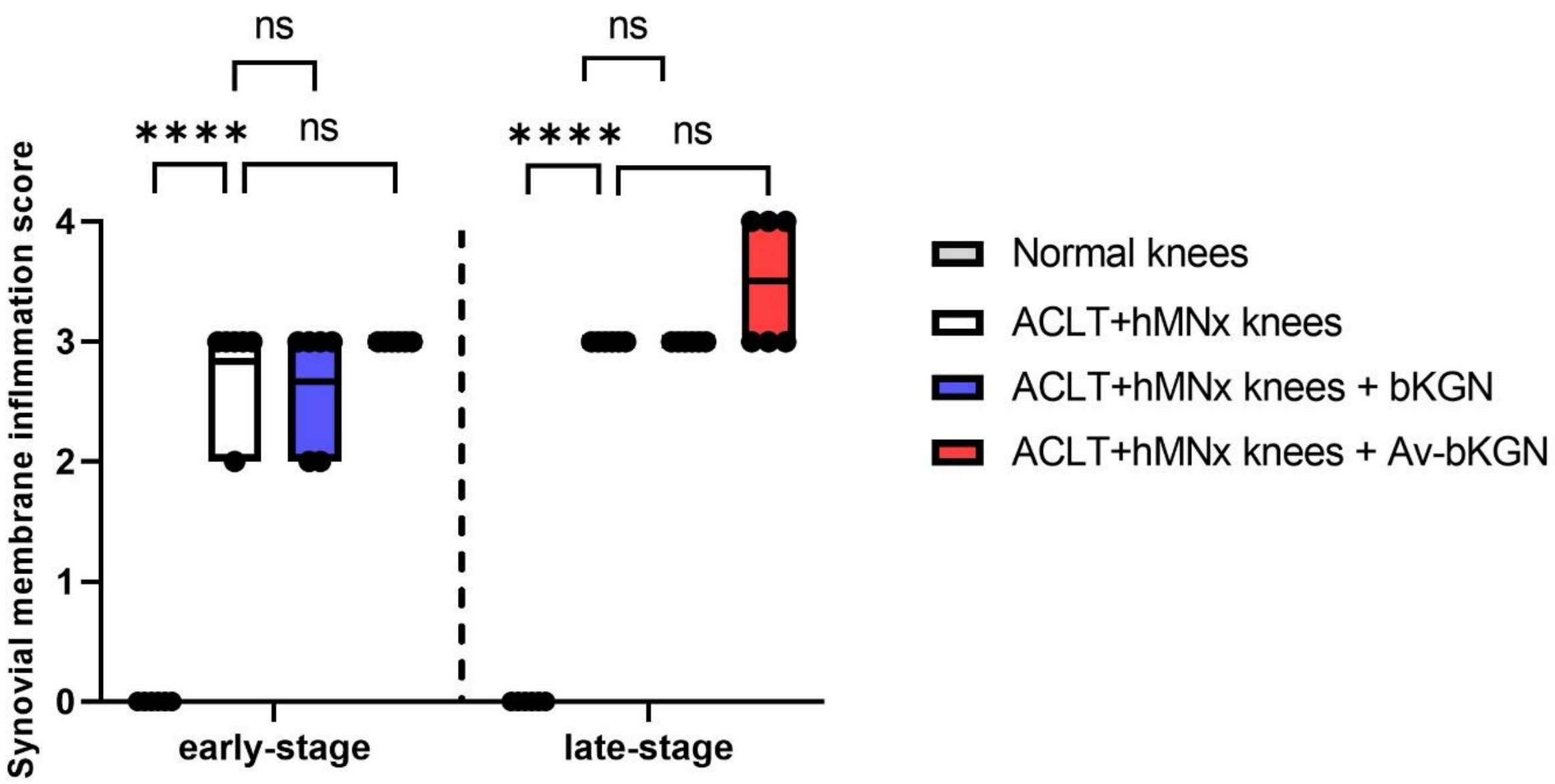

Figure S1. Evaluation of the Synovial membrane inflammation score in the early and late-stage. Statistical comparison between the different groups was realized using an ANOVA Two-Way analysis (Tukey's multiple comparison test) with GraphPad Prism 8.0.1 software (San Diego, CA, USA). Data were reported as the mean ± s.d., considering a 95 % confidence interval at significance level **** p < 0.0001. Groups are resumed as follows for the early-study: *n* = 6 operated right knees without treatment, *n* = 6 contralateral healthy knees of the control group, *n* = 6 operated right knee post-treatment bKGN, *n* = 6 operated right knee post-treatment Av-bKGN. For the late-stage study: *n* = 5 operated right knees without treatment, *n* = 6 contralateral healthy knees of the control group, *n* = 6 operated right knee post-treatment bKGN, *n* = 6 operated right knee post-treatment Av-bKGN.
